# *In silico* identification of potential inhibitors of key SARS-CoV-2 3CL hydrolase (Mpro) *via* molecular docking, MMGBSA predictive binding energy calculations, and molecular dynamics simulation

**DOI:** 10.1371/journal.pone.0235030

**Published:** 2020-07-24

**Authors:** M. Iqbal Choudhary, Muniza Shaikh, Atia- tul-Wahab, Atta- ur-Rahman

**Affiliations:** 1 Dr. Panjwani Center for Molecular Medicine and Drug Research, International Center for Chemical and Biological Sciences, University of Karachi, Karachi, Pakistan; 2 H. E. J. Research Institute of Chemistry, International Center for Chemical and Biological Sciences, University of Karachi, Karachi, Pakistan; University of Calgary, CANADA

## Abstract

The incidence of 2019 novel corona virus (SARS-CoV-2) has created a medical emergency throughout the world. Various efforts have been made to develop the vaccine or effective treatments against the disease. The discovery of crystal structure of SARS-CoV-2 main protease has made the *in silico* identification of its inhibitors possible. Based on its critical role in viral replication, the viral protease can prove to be a promising “target” for antiviral drug therapy. We have systematically screened an *in-house* library of 15,754 natural and synthetic compounds, established at International Center for Chemical and Biological Sciences, University of Karachi. The *in silico* search for potential viral protease inhibitors resulted in nine top ranked ligands (compounds **1**–**9**) against SARS-CoV-2 main protease (PDB ID: 6LU7) based on docking scores, and predictive binding energies. The *in silico* studies were updated *via* carrying out the docking, and predictive binding energy estimation, with a recently reported crystal structure of main protease (PDB ID: 6Y2F) at a better resolution *i*.*e*., 1.95 Å. Compound **2** (molecular bank code AAA396) was found to have highest negative binding energy of −71.63 kcal/mol for 6LU7. While compound **3** (molecular bank code AAD146) exhibited highest negative binding energy of -81.92 kcal/mol for 6Y2F. The stability of the compounds- *in complex* with viral protease was analyzed by Molecular Dynamics simulation studies, and was found to be stable over the course of 20 ns simulation time. Compound **2,** and **3** were predicted to be the significant inhibitors of SARS-CoV-2 3CL hydrolase (Mpro) among the nine short listed compounds.

## 1. Introduction

The recent outbreak of series of pneumonia cases in Wuhan (named as COVID-19 by WHO) has created a medical emergency, unprecedented in recent history. The disease has clinical presentation resembling viral pneumonia, and has emerged as an epidemic [1]. The incident was first reported in central China, in December 2019 [2, 3]. By January 2020, 41 patients were admitted to hospital from which 73% were male with a median age of 49 years. Among them 66% of the patients were found to be exposed to Wuhan seafood market. Common symptoms observed at the onset of illness were cough, fever, and fatigue. All of the 41 patients were positive for pneumonia with abnormal findings on chest CT scan. Acute respiratory distress syndrome, RNAemia, acute cardiac injury, and secondary infection were recorded as complications [4, 5]. Extensive sequencing analysis of the samples from lower respiratory tract identified a virus resembling SARS CoV, and named as novel corona virus 2019 (2019-nCoV) or SARS-CoV-2 [6].

As of 29^th^ May 2020, the number of COVID-19 cases has been reached to 5909,029 with 362,081 deaths reported worldwide. It has been spread in more than 94 countries, including major outbreaks in South Korea, Iran, and Italy [7].

Corona virus belongs to family Coronaviridae, and order Nidovirales. They are enveloped positive sense RNA virus, widely distributed in mammals including humans [8]. Once the cell is infected with SARS-CoV-2, the existing molecular machinery of the host cell is taken over by the virus to translate its RNA into long chains of proteins, and to produce more copies. These long viral proteins are activated when cut into smaller pieces by proteases. Hence, the viral proteases have a critical role in the propagation of the virus. A large number of viral protease inhibitors (such as Amprenavir, atazanavir, darunavir, boceprevir, grazoprevir, etc.) have been approved as antiviral drugs by FDA for the treatment of viral diseases such as HIV, and hepatitis C.

The main protease from SARS-CoV2 has been reported as a heart shape protein, which consist of dimer of identical subunits. The protease activity is triggered by binding of molecules to specific sites. Since 2019-nCov emergence created an urgency for the development of vaccines or effective treatment, various drug repurposing, and virtual screening approaches are being employed in order to find out the possible therapeutic strategy as early as possible. For this purpose, the development of specific inhibitors of the COVID-19 main protease can be of great importance in terms of proposing the treatment regimen.

The gradual evolution of computer hardware, and software technologies have maximized the likelihood of finding the new drugs from huge libraries of small molecules. Through the help of computer-aided drug design techniques, various potential drug targets emerging from genomic and proteomic initiatives can be effectively used to reduce the cost and speed up the drug discovery process. Various studies have employed the use of docking, molecular dynamics simulation, and combination of different advanced *in silico* approaches for drug design [9–11].

In an attempt to address COVID-19 global epidemic challenge, we conducted an *in silico* based virtual screening of an *in-house* chemical library of more than 15,754 natural products and synthetic compounds against the crystal structure of 2019-nCov main protease, also known as 3CL hydrolase (Mpro) submitted in PDB (PDB ID: 6LU7). The enzyme is complexed with an inhibitor, which is peptide in nature. These screened compounds are part of our ICCBS molecular bank, a national chemical repository of Pakistan. The database consists of various natural and synthetic small molecules, having significant biological activities. During this study, we have carried out *in silico* identification of potential inhibitors of COVID-19 main protease (6LU7) *via* molecular docking analysis with the predictive binding energy estimation of the selected ligands. The methodology was also carried out for the recently reported crystal structure of main protease (6Y2F) released on PDB on 4^th^ March 2020 [12] having a better resolution of 1.95 Å. The selected complexes of the viral protease-ligand were subjected to molecular dynamics simulation studies for investigating the stability of the complex formed. Based upon the results we have obtained, these new inhibitors, particularly compound **2**, and **3**, can serve as lead molecules in the discovery of direly needed treatment of COVID-19.

## 2. Material and methods

### 2.1 Protein preparation

For docking studies, the recently submitted crystal structures of SARS-CoV-2 main protease (6LU7 by Jin Z. et al. 2020, and 6Y2F by Zhang L. et al. 2020) in PDB were used. Before commencing docking, the protease structures were subjected to preparation by *Protein Preparation Wizard* in Maestro Schrödinger 12 [13, 14]. During preparation, the missing hydrogens were added, and partial charges were assigned using OPLS-3e force field. Hydrogens and heavy atoms were optimized by restrained minimization.

### 2.2. Ligand preparation

The 2D structures of 15,754 compounds from molecular bank converted to 3D structure, *via Ligprep* module in Maestro Schrödinger 12 [15]. *Ligprep* corrects the protonation, and ionization states of the compounds, and assigned proper bond orders. Afterwards, the tautomeric and ionization states were created for each ligand.

### 2.3. Receptor grid generation and molecular docking

The grid box was defined by selecting the co-crystallized peptide inhibitors (N_3_ and α-ketoamide of 6LU7, and 6Y2F, respectively) to keep the center of each docked ligand with same dimensions of binding box. Rigid receptor docking protocol was run in standard precision (SP) mode of Glide based on OPLS-3e force field [16–18]. During the process of docking, the protein was fixed, while ligands were flexible.

Molecular mechanics-generalized Born surface area (MM-GBSA) method in Prime was used for rescoring the docked pose of ligand [19, 20]. These poses were taken as inputs for the energy minimization of the protein-ligand complexes (E_complex_), the free protein (E_protein_), and the free ligands (E_ligand_). The binding free energy ΔG_bind_ was determined according to the following equation:

ΔGbind=E_complex(minimized)−E_ligand(minimized)−E_receptor(minimized)

### 2.4. ADME properties prediction

The ADME properties, and drug likeness of selected compounds were determined in terms of distribution, absorption, metabolism, excretion, etc., *via* QikProp module of Maestro Schrodinger [21].

### 2.5. Molecular dynamics simulation

MD simulations [22, 23] were performed with Desmond, as implemented in the Schrödinger package. The selected ligand-protein complexes were first immersed into SPC (simple point charge) water box, extending 10 Å beyond any of the complex’s atoms. Counter ions (33 Na^+^, and 29 Cl^-^ ions) were added to neutralize charges. Salt concentration was set to 0.15 M sodium, and chloride ions to approximate physiological condition. The MD was performed in the NPT ensemble at temperature of 300 K and 1.63 bar pressure over 20 ns with recording intervals of 1.2 ps for energy and 20 ps for trajectory. Simulations were run with the OPLS-3e force field. Plots and figures were sketched with Desmond simulation interaction diagram tool of Maestro.

## 3. Results and discussion

### 3.1. Protein and ligand preparation

**Fig 1:**
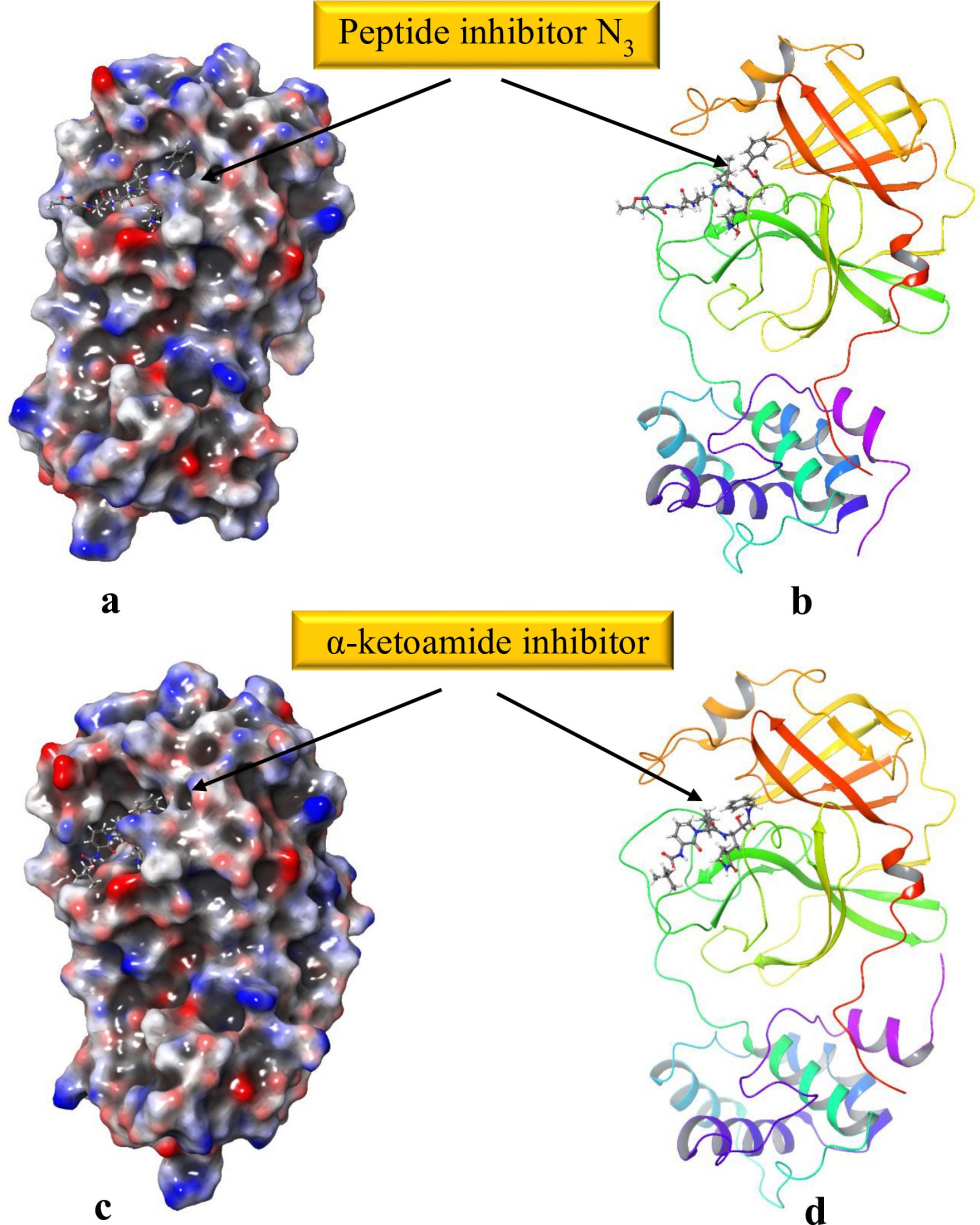
3CL pro hydrolase prepared via *Protein Preparation Wizard*, Maestro. H-bond network optimized and geometry minimized structures of 2019-nCOV 3CL hydrolase (Mpro) PDB ID: **(a)**, and **(b)** 6LU7, **(c)**, and **(d)** 6Y2F represented as 3D molecular surface and ribbon structure.

The conversion of 2D structures to 3D, tautomerization and ionization *via LigPrep* yielded 28,000 minimized 3D molecular structures. These energy minimized 3D structures were used for docking with crystal structure of the 3CL hydrolase (Mpro). Preparation of the viral protease (6LU7, and 6Y2F) by protein preparation wizard tool optimized the H-bond network, and minimized the geometry. The assurance of assigning the proper formal charge, and force field treatment was achieved by adding missing hydrogens and correct ionization states (Fig 1).

### 3.2. Molecular docking studies

**Fig 2:**
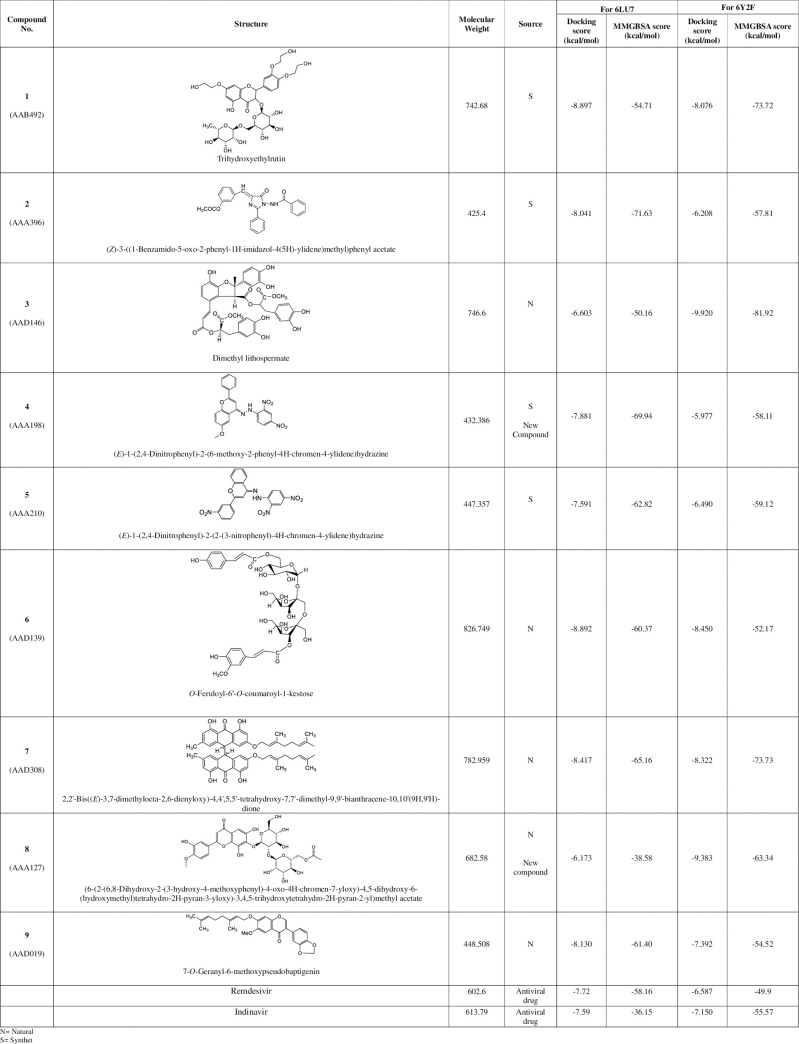
Ligands identified against SARS-CoV-2 3CL protease (6LU7 and 6Y2F) from *in-house* chemical library *via in silico* screening.

After defining the grid box in the prepared viral protease *via Receptor Grid Generation* tool of *Glide* in Maestro, the prepared 3D molecular structures were docked into the co-crystallized inhibitor binding site of viral protease. Fig 2 shows the result of nine docked ligands chosen on the basis of most negative docking score from 15,754 diverse compounds. These scores represent the best-bound ligand conformations, and relative binding affinities. The molecule having the Molecular Bank code AAB492 (compound **1**), and AAD139 (compound **6**), exhibited the highly negative docking score of -8.897, and -8.892 kcal/mol, respectively, while compounds AAA396 (compound **2**), AAD308 (compound **7**), and AAD019 (compound **9**) exhibited a comparable docking score, (ranging from -8.417- -8.04 kcal/mol), and hence inferred to have similar relative binding affinity. AAD146 (compound **3**) and AAA127 (compound **8**), and AAA198 (compound **4**) and AAA210 (compound **5**) were found to have a similar docking score.

The docking analysis was updated in terms of re-docking the selected nine compounds with the recently released crystal structure of 3CL pro hydrolase (PDB ID: 6Y2F) at better resolution of 1.95 Å. Analysis of the docking score of the nine compounds with the α-ketoamide binding site of 2019-nCoV main protease (PDB ID 6Y2F) revealed different docking score, hence binding affinities (Fig 2). These differences can be attributed to the difference in the grid formation due to the presence of α-ketoamide inhibitor in the binding site of 6Y2F. Compounds **1**, **6**, **7**, and **9** exhibited similar docking score as that of 6LU7. Compounds **2**, **3**, **4**, and **5** showed different docking score from docking with 6LU7. However, compound **3** exhibited highly negative docking score of -9.920 kcal/mol against 6Y2F.

Literature survey for the selected compounds was conducted in order to find out their reported activities, mentioned in Table 1.

**Table 1:** Sources of the compounds and reported biological activities of *in silico* identified ligands of SARS-CoV-2 3CL-pro main proteases.

Compound	Source	Reported activities
**1** (AAB492)	Synthetic compound	Antioxidant [24], mild inhibitory activity against lipid peroxidation [25], and antiviral activity against vesicular stromatitis virus (VSV) [26]. Used in the treatment of lupus erythematosus disseminatus [27]
**2** (AAA396)	Synthetic compound	Antioxidant activities [28].
**3** (AAD146)	Found in the roots of *Salvia miltiorrhiza*, *Salvia chinensis*, *Salvia yunnanensis*,*Clerodendranthusspicatus*,and *Salvia przewalskii*	Anti-inflammatory activity [29], suppressesarrhythmogenesis [30], and liver fibrosis/cirrhosis [31], and also act as a Na^+^ channel agonist [32]
**4** (AAA198)	New* synthetic compound	_
**5** (AAA210)	New* synthetic compound	_
**6** (AAD139)	Natural product derived from *Lindelofiastylosa*	*In vitro* immunomodulatory activities [33].
**7** (AAD308)	Natural product extracted from *Psorospermumaurantiacum*, *Cratoxylumformosum*,*Pruniflorum*, *Vismiaguineensis*, *Vismiaorientalis*, *Psorospermumtenuifolium*, and *Psorospermumfebrifugum*.	antiprotozoal activity [34]
**8** (AAA127)	New* compound of natural origin extracted from *Stachys parviflora*	_
**9** (AAD019)	Natural compound derived from *Millettiaconraui*	α-glucosidase inhibitory activity [35]

*The compound has been identified as new

Docking of molecule in protein binding site is a robust approach to elucidate the correct binding pose among several predicted poses of a compound. However, the approach still lacks the ability to correctly rank the affinities of the small molecules to the target protein. In order to have a better ranking of the ligands, and determination of predictive binding energies, MMGBSA calculations were performed. The values obtained after calculation were approximate free energies of binding with a more negative value indicating a stronger binding. Fig 2 also depicts the predictive binding energies of ligand in the binding site of the viral protease. Compound **2** (AAA396) exhibited highest negative binding energy of approximately −71.63 kcal/mole with in the N_3_ binding site of 6LU7 however, compound **3** (AAD146) manifested a predictive binding energy of -81.92 kcal/mole in the α-ketoamide binding site of 6Y2F.

**Fig 3:**
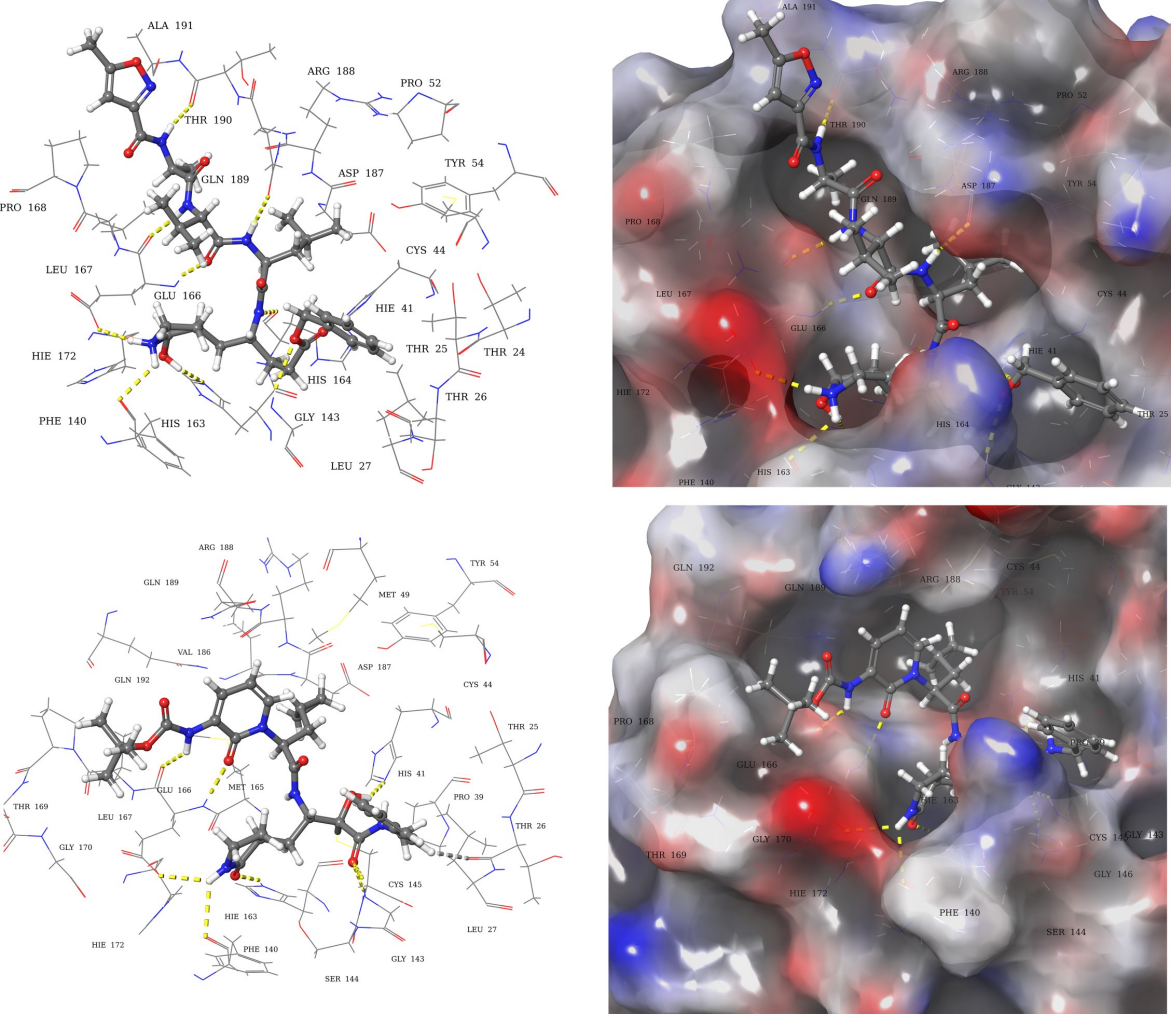
Molecular interactions of 3CL pro hydrolase with co-crystallized inhibitors. (**a**) 3D-Ligand interaction and (**b**) 3D molecular surface diagrams of co-crystallized peptide inhibitor in the binding site of 6LU7. (**c**) 3D-Ligand interaction and (**d**) 3D molecular surface diagrams of co-crystallized α-ketoamide inhibitor in the binding site of 6Y2F. The hydrogen bonds are represented as yellow dotted lines.

Analysis of the co-crystallized peptide inhibitors (N_3_, and α-ketoamide) revealed their molecular interaction with the binding site of viral proteases (6LU7, and 6Y2F, respectively). The peptide inhibitor N_3_ interacted through hydrogen bonds with residues Thr190, Gln189, Glu166, His164, Phe140, and Gly143 (Fig 3A) of 6LU7 with the depiction of binding site occupancy in 3D molecular surface diagram (Fig 3B). The α-ketoamide inhibitor also interacted through hydrogen bonds in the binding site of 6Y2F with residues His41, Gly143, Cys145, His163, His164, and Glu166 (Fig 3C and 3D).

Molecular recognition of viral protease-ligand complex at atomic level was also determined by analyzing the bound conformation of the ligands. The finally selected pose of the ligands, based on docking score and predicted binding energy, were studied to decipher the molecular interactions of viral protease (6LU7, and 6Y2F) with docked ligands.

**Fig 4:**
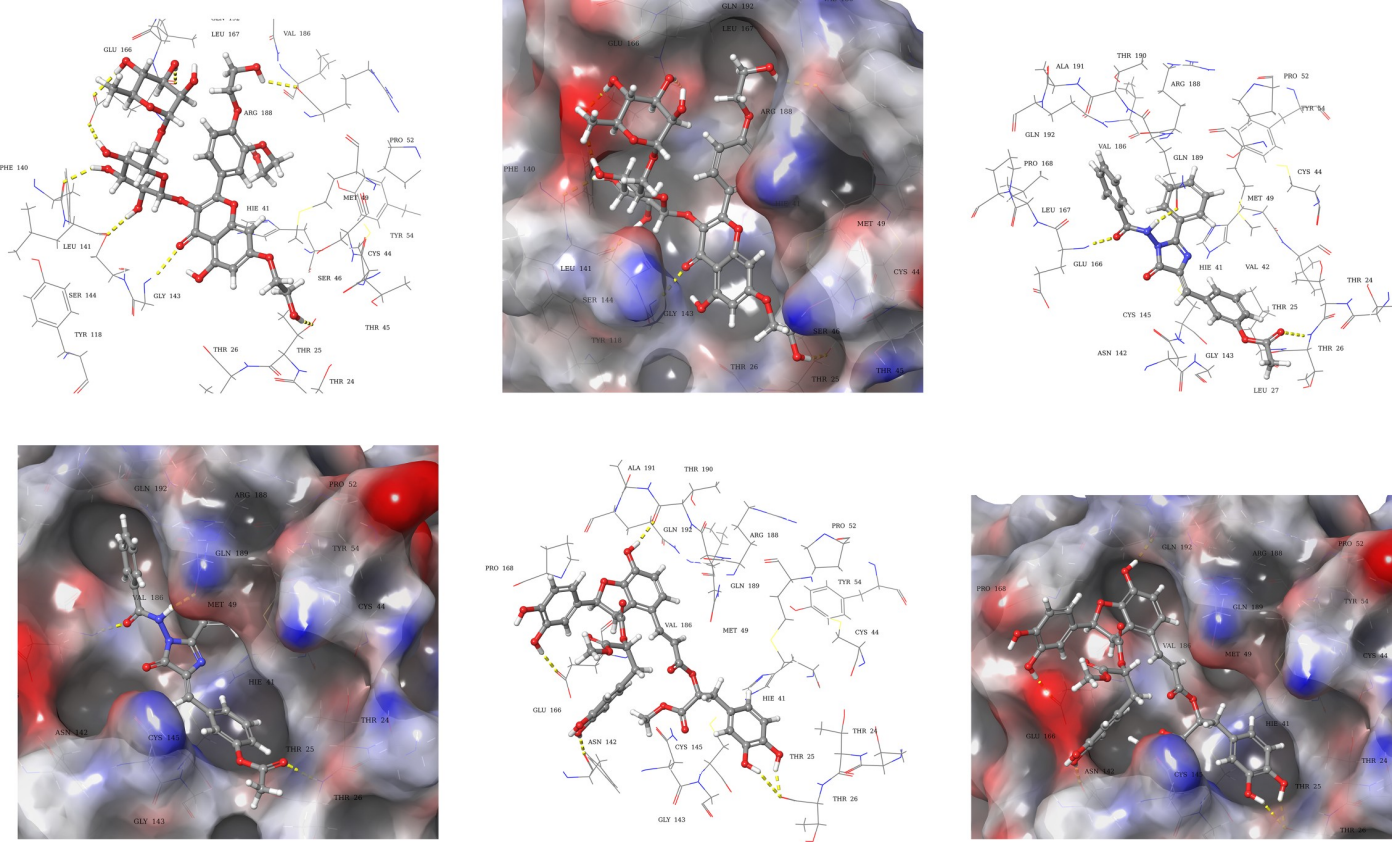
Docked poses and molecular interactions of compound 1, 2, and 3 in binding site of 6LU7. 3D-Ligand interaction and 3D molecular surface diagrams of compounds **1** (**a**) and (**b**), compound **2** (**c)** and (**d**) compound **3** (**e**) and (**f**), showing the docked pose in the binding site of 2019-nCOV 3CL hydrolase (Mpro). The hydrogen bonds are represented as yellow dotted lines.

Compound **1** (AAB492) interacted through hydrogen bonds (Fig 4A) with the binding site residues of SARS-CoV-2 main protease (6LU7). The binding site residues Thr45, Leu141, Gly143, Glu166, and Arg188, of viral protease exhibited hydrogen bonding with the various hydroxyl groups of compound **1**. Compound **2** (AAA396) showed interactions with residues Gln189, Glu166, and Thr26 *via* hydrogen bonds (Fig 4C). Compound **3** (AAD146) adopted a different binding pose, the interactions made between viral protease binding site, and compound **3** (AAD146) were through similar residues as that of N_3_ peptide inhibitor, *i*.*e*., hydrogen bonds with residues Thr26, Phe140, Glu166, and Thr190 (Fig 4E). The occupancy of the binding site can be observed in 3D molecular surface diagrams (Fig 4B, 4D and 4F). The compounds well accommodated the binding site with fully occupying the binding pocket. The docking analysis led to the prediction that the compounds are interacting with the SARS-CoV-2 hydrolase binding site through same residues as that of co-crystallized inhibitor.

**Fig 5:**
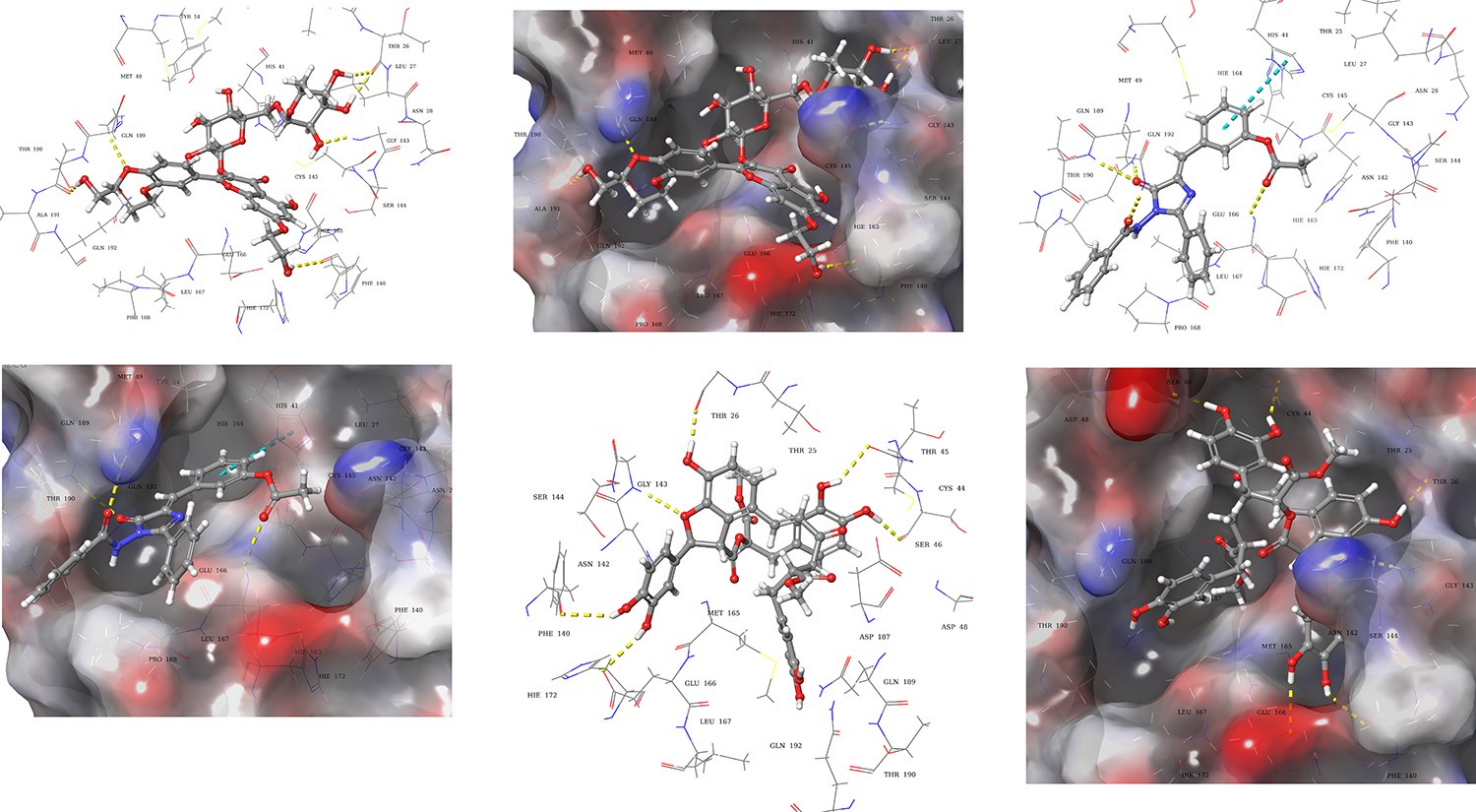
Docked poses and molecular interactions of compound 1, 2, and 3 in binding site of 6Y2F. 3D-Ligand interaction and 3D molecular surface diagrams of compounds **1** (**a**) and (**b**), compound **2** (**c)** and (**d**) compound **3** (**e**) and (**f**), showing the docked pose in the binding site of 2019-nCOV 3CL hydrolase (Mpro). The hydrogen bonds, and π-π interaction are represented as yellow and blue dotted lines.

Molecular interactions and binding site occupancy of compound **1**, **2**, and **3** in the α-ketoamide binding site of 6Y2F were also analysed. Compound **1** (AAB492) made molecular interactions with residues Thr26, Phe140, Gly143, Gln189, and Thr26 through hydrogen bonding (Fig 5A and 5B). Compound **2** (AAA396) showed hydrogen bonding with Thr190, Gln192, Gln189, and Glu166. In addition to this a π-π interaction with His41 was also observed (Fig 5C and 5D). Compound **3** (AAD146) exhibited hydrogen bonding with residues Thr26, Cys44, Ser46, Glu166, Phe140, and Gly143

Comparison of the molecular docking results of reported antivirals for COVID-19 *i*.*e*., remdisivir, and indinavir with 6LU7, and 6Y2F was also carried out. The analysis revealed comparable docking score (Fig 2) as that of 9 selected compounds. However, the predicted binding energies were found to be lower as compared to the selected hits.

A summary of type of contacts between the nine ligands and biding site residues of 6LU7, and 6Y2F has been given as S1 Table in supporting information.

### 3.3. *In silico* ADME properties of selected ligands

Table 2 entails the ADME (absorption, distribution, metabolism, and excretion) properties predicted *via* QikProp. The ADME analysis determines the physicochemical properties and biological functions as well as drug likeness of the compound. This is important in terms of assessing the efficacy of drug molecules. The descriptors such as molecular weight, drug-likeness, dipole moment, solvent accessible surface area, hydrogen bond donor, and acceptor traits, octanol/water coefficient, aqueous solubility, binding to human serum albumin, number of likely metabolic reactions, brain/blood partition coefficient and human oral absorption were predicted for compound **2**, and **3** and compared with reported antiviral drugs indinavir, and remdesivir. The values obtained for compound **2** are in recommended range. Compound **2** exhibited better pharmacokinetic descriptors as compared to clinically approved antiviral drugs in terms of molecular weight drug-likeness, and human oral absorption. However the ADME analysis of compound **3** suggests that further derivatization in molecular structure can be done to improve the molecular descriptors such as molecular weight, acceptor hydrogen bond, number of likely reactions, brain/blood partition coefficient and % human oral absorption. This will eventually lead to an improvement in the drug-likeness score (# star) of the compound **3**.

**Table 2:** *In silico* predicted ADME properties of compounds 2, and 3.

**Compound**	Mol. Wt.	#Stars	Dipole	SASA	Donor H-bond	Acceptor H-bond	QPlogPo/w	QPlogS	QPlogkhsa	No. of Metabolites	QplogBB	% Human oral Absorption
**2**	425.4	2	6.56	748	0	8	3.7	-5.7	0.252	0	-1.28	100
**3**	746.6	11	9.749	103	7	14	2.643	-6.110	0.254	13	-5.723	0.5
Indinavir	613.7	6	5.549	1017	4	13	2.787	-3.866	-0.235	12	-0.724	59.6
Remdesivir	602.5	5	12.706	884	5	16	1.271	-4.856	-0.590	6	-3.278	34.504
Recommen-ded range	130–725	0–5	1.0–12.5	300–1000	0–6.0	2.0–20.0	-2-6.5	-6.5–0.5	-1.5–1.5	1–8	-3-1.2	>80% is high <25% is poor

**Key**:

**#Stars:** Number of property or descriptor values that fall outside the 95% range of similar values for known drugs. A large number of stars suggests that a molecule is less drug-like than molecules with few stars

**Dipole:** Computed dipole moment of the molecule.

**SASA:** Total solvent accessible surface area (SASA) in square angstroms using a probe with a 1.4 Å radius.

**Donor H-bond:** Estimated number of hydrogen bonds that would be donated by the solute to water molecules in an aqueous solution.

**Acceptor H-bond:** Estimated number of hydrogen bonds that would be accepted by the solute from water molecules in an aqueous solution.

**QPlogPo/w:** Predicted octanol/water partition coefficient.

**QPlogS:** Predicted aqueous solubility, log S.

**QPlogkhsa:** Prediction of binding to human serum albumin

**No. of Metabolites:** Number of likely metabolic reactions.

**QplogBB:** Predicted brain/blood partition coefficient.

**% Human Oral Absorption:** Predicted human oral absorption on 0 to 100% scale.

### 3.4. Molecular dynamics simulation with Desmond package

Since docking is a static view of the binding pose of molecule in the active site of protein, Molecular dynamics (MD) simulation tend to compute the atom movements with time by integrating the Newton’s classical equation of motion [36]. In MD, the dynamic behavior of the molecular system is simulated to assess the stability of protein ligand complex. Therefore, the docked conformers of compound **2** (AAA396), and **3** (AAD146) with highest predictive binding energy of −71.63 and -81.92 kcal/mol, repectively, were used for molecular dynamics study with OPLS3e force field.

**Fig 6:**
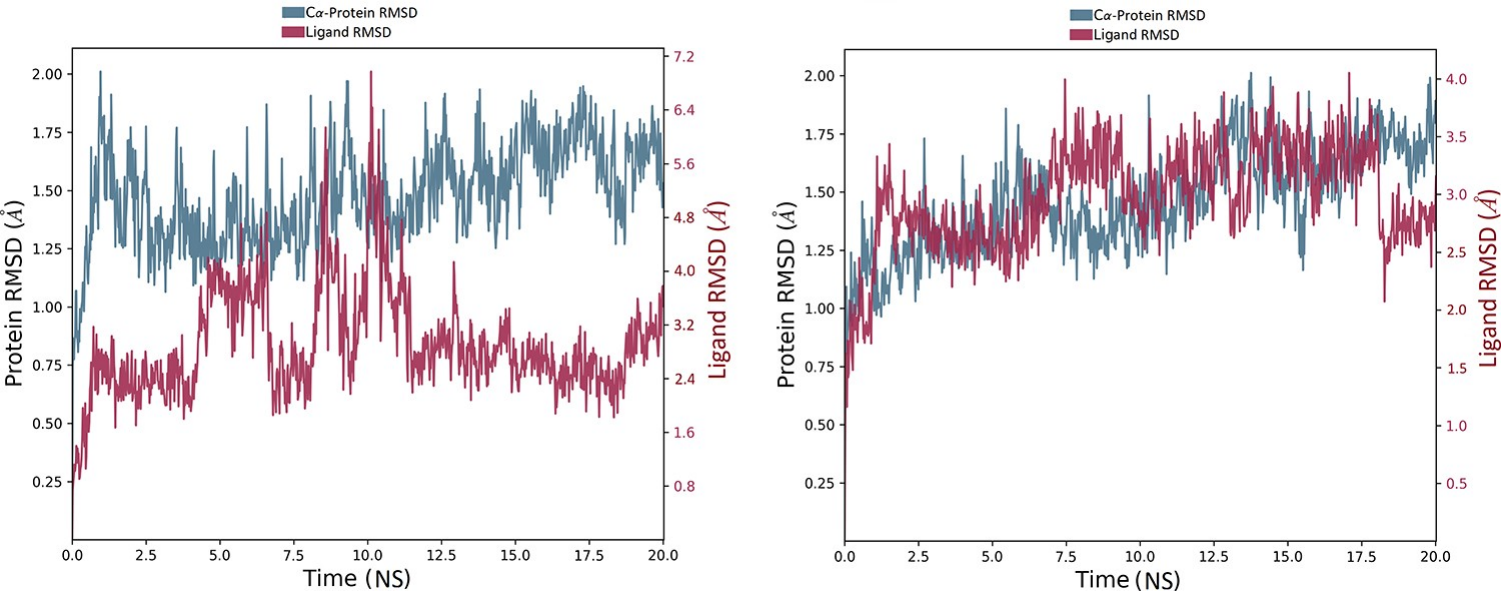
RMSD analysis of MD simulation trajectory. The RMSD plot obtained for **(a)** compound **2**-SARS-CoV-2 main protease complex (PDB ID 6LU7), and **(b)** compound **3**-SARS-CoV-2 main protease complex (PDB ID 6Y2F). The simulation time of 20 ns showing the formation of stable complex without any significant conformational changes in protein structure.

The root mean square deviation (RMSD) plot in Fig 6A indicates that the compound **2**-SARS-CoV-2 main protease (PDB ID 6LU7) complex stabilized shortly after commencing the simulation *i*.*e*., 2.5 ns, with respect to the reference frame at time 0 ns. The plot showed the RMSD of viral protease on left Y-axis, while the Y-axis on right shows the ligand RMSD profile aligned on protein backbone. All the frames from 20 ns trajectory are aligned on reference frame backbone. In case of ligand RMSD, the fluctuation was observed during first 2 ns of trajectory. However, the variations were in order of 0.15 Å showing that the complex has not undergone large conformational changes. Furthermore, after 7 ns the simulation starts showing fluctuations in ligand RMSD of order of 2.8 Å for up to 12 ns. The ligand RMSD stabilized again after 12 ns which remain consistent till the end of simulation time of 20 ns.

The protein RMSD tend to be stable throughout the simulation with insignificant fluctuations ranging between 0.5–1.0 Å implying that the protease has not undergone large conformational changes. The overall RMSD plot of compound **2**-SARS-CoV-2 main protease complex shows fluctuations of 1.45 Å suggesting that the ligand is stably bound to protease binding site and has not diffused away from the bound position.

The RMSD plot in Fig 6B indicates the compound **3**-SARS-CoV-2 main protease (PDB ID 6Y2F) complex MD trajectory of 20 ns. The complex tends to be stabilized during the course of simulation with respect to reference frame at time 0 ns. A slight divergence can be seen towards the end of the simulation *i*.*e*., 18 ns. However, the fluctuation lies under the permissible range of 1–3 Å, hence, can be considered as non-significant. Since the RMSD plots of compound **3** and protein backbone were lying over each other, formation of a stable complex can be inferred.

**Fig 7:**
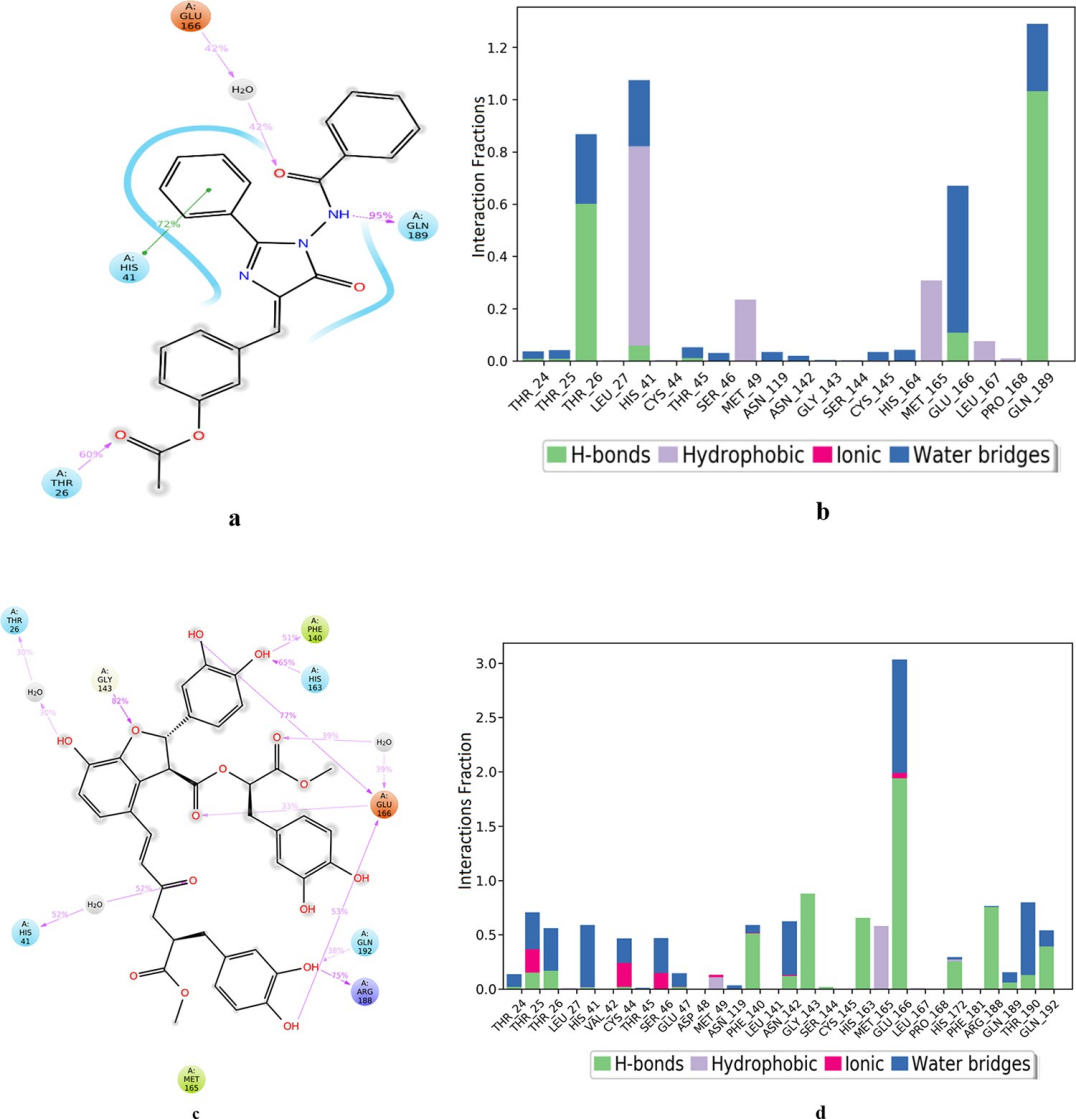
Analysis of molecular interaction and type of contacts with 3CL pro hydrolase after MD simulation. Detailed schematic interaction of **(a)** compound **2** (AAA396), and **(c)** compound **3** atoms with binding site residue of hydrolase crystal structures 6LU7, and 6Y2F, respectively. Interaction happening more than 30% of the simulation time are shown. Normalized stacked bar chart of viral protease binding site residues interacting with **(b)** compound **2** and **(d)** compound **3** *via* hydrogen bond, hydrophobic and ionic interactions, and water bridges.

Fig 7A shows the residue interactions of viral protease with compound **2** (AAA396). Interactions that last more than 30% of the simulation time were considered. It shows that all of the interactions of docked pose were retained during the simulation time of 20 ns *i*.*e*. molecular interactions with residues Glu166, Gln189, and Thr26. Moreover, a hydrophobic contact in the form of π-π interaction was also established between the His41 and benzene moiety of compound **2**. Fig 7B shows the viral protease-compound **2** (AAA396) contacts in the form of stacked bar charts which are normalized over the course of 20 ns trajectory. These contacts are categorized into hydrogen bonds, hydrophobic, and ionic interactions, and water bridges. The bar chart shows that hydrogen bonds, hydrophobic contacts, and water bridges prevailed during the course of simulation. The important hydrogen bonds observed in the initial docked pose of compound **2** (Thr26, Glu166, Gln189) did not change during the MD simulation. Hydrogen bonding with residue Gln189 retained for more than 100% of the simulation time. This might be due to the formation of multiple hydrogen bonds of same subtypes *i*.*e*, backbone acceptor; backbone donor; side-chain acceptor; side-chain donor. The same residues were also involved in forming water bridged interactions. Hydrophobic interactions were also observed with residues His 41, Met149, and Met165, which lasted for more than 30% of the simulation time. Water bridged interactions were also observed with residues Thr 24, Thr 25, Thr 26, Thr 45, Thr 46, Asn 119, Asn 142, Cys145, His 164, Glu 166, and Gln 189. However, the water bridges with Thr26, and Glu 166 last for 30% of the simulation. Hence, the four residues Thr 26, His 41, Glu 166, and Gln 189 were identified as the key residues involved in formation of 6LU7-compound **2** complex.

The fractional residue interactions of the α-ketoamide binding site of 6Y2F with compound **3** is presented in Fig 7C showing the interactions that persist for at least 30% of the simulation time. The interactions with residue Thr26, Glu166, Phe 140, and Gly143 were retained from the docked pose. However, new interactions were also established with residues His41, His163, Arg188, and Gln192. Fig 7D shows the types of viral protease-compound **3** (AAD146) contacts that were formed during 20 ns MD simulation. It can be seen that the interactions were of various types *i*.*e*., hydrogen bonds, water bridges, ionic, and few of them were hydrophobic. The cumulative fractional time for interactions made by most of the residues was 30–50% of total simulation time of 20 ns. However Glu166 showed interaction for more than 100% of the simulation time due to the simultaneous formation of interactions of same subtypes. This can be attributed to the predicted ADME properties of compound **3** depicted in Table 1. According to ADME analysis the compound **3** is capable of donating 7, and accepting 14 hydrogen bonds with the water molecules of the aqueous solution. Compound **3** also exhibited a very high solubility profile evident from its QPlogS value of -6.511. Glu166, Asn142, Arg188, Thr190 and Gly143 of 6Y2F were found to be critically important since they interacted with compound **3** for more than 70% of the total simulation time.

## 4. Conclusion

From the present study, it can be inferred that the computational methods can efficiently identify the inhibitors of a key enzyme/ protein of SARS-CoV-2, which is currently a global epidemic. The results need to be subjected to *in vitro* studies for further validation. The study identified nine compounds from an *in-house* chemical library of natural and synthetic compounds, chosen on the basis of docking score and predicted binding energies. Compound **2** (AAA396) and compound **3** (AAD146) are predicted to be the potential inhibitors of 2019-nCoV main protease. However, derivatization of compound **3** is recommended to improve its ADME profile. In view of the fact that no treatment remedy has been devised so far for corona virus infection, the data presented here can be helpful in identifying the lead molecule for future *in vitro* and *in vivo* studies and for the drug discovery and development against COVID-19.

## Supporting information

S1 Table Summary of the contacts and interacting residue of SARS-CoV-2 3CL protease (6LU& and 6Y2F) with the docked compounds. (DOCX)
