# Supplementary material for: In silico identification of potential inhibitors of key SARS-CoV-2 3CL hydrolase (Mpro) via molecular docking, MMGBSA predictive binding energy calculations, and molecular dynamics simulation
Source: PLoS One. 2020 Jul 24;15(7):e0235030. doi: 10.1371/journal.pone.0235030 (PMC7380638; doi:10.1371/journal.pone.0235030)
Supplement: S1 Table — (DOCX) [file pone.0235030.s001.docx]

**Table S1**: Summary of the contacts and interacting residue of SARS-CoV-2 3CL protease (6LU& and 6Y2F) with the docked compounds

| **Compounds** | **Interacting Residue** | **Type and No. of Contacts** | **Distances (Å)** | **Vander Waals contacts radii** |
| --- | --- | --- | --- | --- |
| **6LU7** | | | | |
| AAB492  (**1**) | Thr25  Leu141  Gly143  His164  Glu166  Arg188 | H-bond  H-bond  H-bond  H-bond  Four H-bonds  H-bond | 1.88  1.98  2.13  2.11  2.43; 2.14;1.79; 1.71  2.31 | -0.714  -1.821  -1.324  -2.152  -6.233  -1.780 |
| AAA396  (**2**) | Thr26  Glu166  Gln189 | H-Bond  H-Bond  H-Bond | 2.33  1.88  1.86 | -3.097  -2.178  -8.332 |
| AAD146  (**3**) | Thr24  Thr26  Glu166 | H-Bond  H-Bond  H-Bond | 1.92  2.12  1.75 | -1.90  -0.975  -2.336 |
| AAA198  (**4**) | His41  Glu166  Gln192 | π-π stacking  H-Bond  H-Bond | 4.73 | -1.926  -6.612  -2.369 |
| AAA210  (**5**) | Arg188  Gln192 | H-Bond  H-Bond | 2.27  2.06 | -1.241  -2.918 |
| AAD139  (**6**) | Thr25  Phe140  Asn142  Glu166  Gln189  Thr190 | H-Bond  H-Bond  H-Bond  H-Bond  Two H-Bonds  H-Bond | 2.12  1.97  2.10  2.09  2.10; 2.22  2.71 | -0.631  -0.467  -3.957  -5.149  -8.718  -0.879 |
| AAD308  (**7**) | His41 | π-π stacking | 5.32 | -3.785 |
| AAA127  (**8**) | Thr24  Thr26  Cys145  Glu166  Gln189 | H-Bond  H-Bond  H-Bond  Three H-Bonds  H-Bond | 2.37  2.63  2.45  1.74; 2.14; 1.79  2.66 | -1.70  -1.862  -1.238  -4.379  -3.311 |
| AAD019  (**9**) | Thr26  Gly143  Glu166 | H-Bond  H-Bond  H-Bond | 1.88  2.07  2.11 | -0.469  -1.715  -4.197 |
| Remdisivir | Gly143  Cys145  Glu166  Leu167  Gln189 | H-Bond  H-Bond  H-Bond  H-Bond  H-Bond | 1.81  2.26  1.85  2.59  1.67; 2.00 | -0.966  -2.191  -4.542  -1.808  -2.782 |
| Indinavir | His41  Asn142  Glu166  Gln189 | π-π stacking  H-Bond  Three H-Bonds  H-Bond | 3.88  1.94  1.99; 1.56; 1.84  2.54 | -4.059  -2.481  1.41  -6.643 |
| **6Y2F** | | | | |
| AAB492  (**1**) | Thr26:OH  Gly143:OH  Glu166:OH  Gln189:OR  Thr190:OH | Two H-bonds  H-bond  H-bond  H-bond  H-bond | 2.31; 2.02  2.12  1.89  2.12  1.97 | -1.315  -1.238  -8.392  -4.193  -1.392 |
| AAA396  (**2**) | His41  Glu166  Thr190  Gln192 | π-π stacking  H-bond  H-bond  H-bond | 5.49  2.35  2.21  1.79 | -1.588  -4.116  -3.366  -2.663 |
| AAD146  (**3**) | Thr26:OH  Ser46:OH  Gly143:OR  His163:OH  Glu:166:OH | H-bond  H-bond  H-bond  H-bond  Two H-bonds | 1.99  2.23  2.31  2.15  2.77; 2.37 | -1.338  0.119  -1.577  -3.404 |
| AAA198  (**4**) | His41  Gly143:OR  His163:NO_2_  Glu166:NO_2_ | π-π stacking  H-bond  H-bond  Salt bridge | 5.14  2.15  2.43  3.82 | -3.350  -1.366  -1.606  -4.992 |
| AAA210  (**5**) | His163:NO_2_  Glu166:NO_2_ | H-bond  Salt bridge | 2.18  4.01 | -1.610  -4.073 |
| AAD139  (**6**) | Thr24:OH  His41  Leu141:OH  Asn142:OH  Gly143:CO  His163:OH  Glu166:OH | H-bond  π-π stacking  H-bond  H-bond  H-bond  H-bond  Two H-bonds | 1.81  5.11  2.06  2.31  2.27  2.55  1.67; 1.83 | -2.861  -5.046  -5.952  -1.616  -1.334  0.175 |
| AAD308  (**7**) | His41  Gly143:OH  His164:OH | π-π stacking  H-bond  H-bond | 4.07  1.75  1.72 | -5.592  1.130  0.919 |
| AAA127  (**8**) | Thr26:OH  His41:OH  Asn142:OH  Gly143:OH  Cys145:OH  His163:CO  His164:OH  Glu166:OH | H-bond  H-bond  H-bond  H-bond  H-bond  H-bond  H-bond  H-bond | 1.97  2.04  1.87  2.34  1.99  1.81  1.83  1.95 | -1.333  -5.082  -4.733  -1.745  -2.352  -0.529  -1.697  -3.740 |
| AAD019  (**9**) | Thr26:OR  Gly143:CO  Glu166:OR | H-bond  H-bond  H-bond | 2.32  1.95  1.87 | -3.416  -1.020  -3.408 |
| Remdisivir | Cys145:CO  Glu166  His163:N | H-bond  Two H-bonds  H-bond | 2.39  2.30; 2.80  2.40 | -1.202  -6.344  -1.641 |
| Indinavir | His164:NH  Gln192:N | H-bond  H-bond | 2.68  2.75 | -3.065  -2.367 |
